# Preservation of epoxyeicosatrienoic acid bioavailability prevents renal allograft dysfunction and cardiovascular alterations in kidney transplant recipients

**DOI:** 10.1038/s41598-021-83274-1

**Published:** 2021-02-12

**Authors:** Thomas Duflot, Charlotte Laurent, Anne Soudey, Xavier Fonrose, Mouad Hamzaoui, Michèle Iacob, Dominique Bertrand, Julie Favre, Isabelle Etienne, Clothilde Roche, David Coquerel, Maëlle Le Besnerais, Safa Louhichi, Tracy Tarlet, Dongyang Li, Valéry Brunel, Christophe Morisseau, Vincent Richard, Robinson Joannidès, Françoise Stanke-Labesque, Fabien Lamoureux, Dominique Guerrot, Jérémy Bellien

**Affiliations:** 1 Department of Pharmacology, Rouen University Hospital, 76000 Rouen, France; 2 UNIROUEN, INSERM U1096, FHU CARNAVAL, Normandie University, 76000 Rouen, France; 3 Laboratory of Pharmacokinetics, Toxicology and Pharmacogenetics, Rouen University Hospital, 76000 Rouen, France; 4 Department of Nephrology, Rouen University Hospital, 76000 Rouen, France; 5 Department of Pharmacology, Grenoble Alpes University Hospital, HP2, INSERM U1042, University of Grenoble Alpes, 38000 Grenoble, France; 6 Department of Entomology and Nematology, and Comprehensive Cancer Center, University of California, Davis, Davis, CA 95616 USA; 7 Department of General Biochemistry, Rouen University Hospital, 76000 Rouen, France; 8 Centre d’Investigation Clinique (CIC)-INSERM 1404, Rouen University Hospital, 76000 Rouen, France; 9 Department of Pharmacology, Rouen University Hospital, 76031 Rouen Cedex, France

**Keywords:** Diseases, Nephrology

## Abstract

This study addressed the hypothesis that epoxyeicosatrienoic acids (EETs) synthesized by CYP450 and catabolized by soluble epoxide hydrolase (sEH) are involved in the maintenance of renal allograft function, either directly or through modulation of cardiovascular function. The impact of single nucleotide polymorphisms (SNPs) in the sEH gene *EPHX2* and *CYP450* on renal and vascular function, plasma levels of EETs and peripheral blood monuclear cell sEH activity was assessed in 79 kidney transplant recipients explored at least one year after transplantation. Additional experiments in a mouse model mimicking the ischemia–reperfusion (I/R) injury suffered by the transplanted kidney evaluated the cardiovascular and renal effects of the sEH inhibitor *t*-AUCB administered in drinking water (10 mg/l) during 28 days after surgery. There was a long-term protective effect of the sEH SNP rs6558004, which increased EET plasma levels, on renal allograft function and a deleterious effect of K55R, which increased sEH activity. Surprisingly, the loss-of-function CYP2C9*3 was associated with a better renal function without affecting EET levels. R287Q SNP, which decreased sEH activity, was protective against vascular dysfunction while CYP2C8*3 and 2C9*2 loss-of-function SNP, altered endothelial function by reducing flow-induced EET release. In I/R mice, sEH inhibition reduced kidney lesions, prevented cardiac fibrosis and dysfunction as well as preserved endothelial function. The preservation of EET bioavailability may prevent allograft dysfunction and improve cardiovascular disease in kidney transplant recipients. Inhibition of sEH appears thus as a novel therapeutic option but its impact on other epoxyfatty acids should be carefully evaluated.

## Introduction

While the incidence of acute allograft rejection has decreased over the last decades, chronic allograft nephropathy has only been marginally improved and represents the major cause of long-term dysfunction in kidney transplantation^1–3^. Ischemia–reperfusion injury, innate and adaptive immune activation and calcineurin inhibitors promote vascular toxicity and renal endothelial dysfunction which play a key role in chronic allograft nephropathy^4–6^. In addition, cardiovascular disease remains a leading cause of allograft loss and mortality in kidney transplant recipients^7^. Therefore, the development of new therapeutic strategies that could preserve kidney allograft function directly and/or through vascular protection is of crucial importance.

Epoxyeicosatrienoic acids (EETs) are arachidonic acid derived eicosanoids synthesized by cytochrome P450 enzymes (CYP450) in endothelial and renal cells that induce vasodilation, natriuresis and prevent inflammation and apoptosis^8–11^. After their synthesis, EETs are metabolized to the less active dihydroxyeicosatrienoic acids (DHETs) by the ubiquitous enzyme soluble epoxide hydrolase (sEH), encoded by the *EPHX2* gene^8–11^. Inhibitors of sEH have thus been developed to increase EET bioavailability and protective effects were observed in animal models of diseases^8–11^. Two sEH inhibitors moved to the first phases of clinical development, showing no toxicity^12,13^, but still remain unavailable for human use.

*CYP450* and *EPHX2* genes are highly polymorphic and some single nucleotide polymorphisms (SNPs) are known modulators of their enzymatic activities towards EET biosynthesis and degradation at least in vitro^11^. The determination of the effects of these SNPs on patients’ clinical characteristics represents a common way to assess the interest of modulating EET pathway in disease. To date, only the presence of the *EPHX2* 3-untranslated region (3′-UTR) polymorphism of the recipient was identified as a modifying factor of kidney allograft function^14,15^. However, the controversial results reported are difficult to interpret especially since the effect of this SNP on sEH expression or activity remains unclear^14,15^. In contrast, no impact of the frequent *EPHX2* exonic SNPs i.e., R287Q and K55R that are known to affect sEH activity, has been observed in these studies^14,15^. The reasons for these unexpected findings are not known but simultaneous presence of different *EPHX2* SNPs with opposite effects on sEH activity could be evoked^15,16^. Furthermore, although the modifications of the bioavailability of immunosuppressive agents induced by *CYP450* SNPs have been extensively investigated^17^, only one study suggested that the presence of *CYP450* loss-of-function (LoF) polymorphisms of the donors worsened the outcome of renal transplantation^18^. Moreover, no study has evaluated the impact of *EPHX2* and *CYP450* SNPs on vascular function in kidney transplant recipients.

Thus, the aim of this study was to carefully assess whether genetic variations in *EPHX2* and *CYP450* of kidney transplant recipients affect renal and vascular function taking into consideration their effects on EET metabolism. Moreover, experiments in a mouse model mimicking the ischemia–reperfusion (I/R) injury suffered by the transplanted kidney were performed to directly evaluate the cardiovascular and renal effects of blocking sEH in this context.

## Methods

### Population

A total of 79 patients with a kidney transplantation performed in Rouen University Hospital and followed since at least 1 year were included in this study. Medical records of these patients were reviewed to obtain age at transplantation, donor age, immunosuppressive agents and concomitant treatments, comorbidities and plasma creatinine at 3, 6 and 12 months after kidney transplantation, allowing the determination of estimated glomerular filtration rate (eGFR) using the MDRD equation. All patients were Caucasians in order to limit ethnic variability regarding genetic investigations and aged between 18 and 75 years old. Patients with a previous renal graft or acute renal rejection demonstrated by kidney biopsy were excluded from the study. The study was approved by the local Ethics Committee (Committee for the Protection of Persons of Normandy), and all participants gave written informed consent. The study was conducted in accordance with the Principles of Good Clinical Practice and the Declaration of Helsinki and was registered at https://clinicaltrials.gov under the unique identifier NCT02555566.

### Exploration visit

#### Functional parameters

Measurements were performed in the morning after a fat-free breakfast without tea or coffee while subjects were in a supine position, in a quiet air-conditioned and thermostated room (22–24 °C). Subjects were asked to refrain from smoking from the previous evening. Blood pressure and heart rate were measured on the dominant arm using a brachial cuff oscillometric device (OMRON HEM-705CP). Radial internal diameter and blood flow were measured on the non-dominant arm, using a multiarray high-resolution echotracking and Doppler (ArtLab system, ESAOTE PIE MEDICAL)^19,20^.

Radial artery endothelium-dependent flow-mediated dilatation was assessed during a progressive and sustained increase in blood flow induced by hand skin heating^21–23^. The hand skin temperature was modified by use of a water-filled thermo-controlled device (Polystat 1, BIOBLOCK SCIENTIFIC, Illkirch, France). Briefly, the hand was introduced in the thermo-controlled tank by use of a thin watertight glove fixed to the device. The device was then filled with water and the temperature was fixed to 34 °C for 20 min. Then, hand skin heating was performed by gradually increasing the water temperature from 34 to 44 °C with each level of temperature maintained for 7 min. Radial artery endothelium-independent dilatation was assessed using 0.3 mg sublingual glyceryl trinitrate (GTN, TEOFARMA SRL., Pavia, Italy).

#### Biological parameters

A 4-F catheter was inserted into the forearm cephalic vein, when accessible, allowing blood sampling in the venous return at 34 and 44 °C. Blood sampling was drawn at 34 °C to measure plasma creatinine and to perform *EPHX2* and *CYP450* genotyping from whole blood. Genomic DNA was isolated using a QIamp DNA Blood Mini Kit (QIAGEN, Les Ulis, France) according to manufacturer’s instructions. Genotyping was carried out by Taqman allelic discrimination assays (THERMO FISHER SCIENTIFIC, Courtaboeuf, France) on a 7500 Fast Real-Time PCR System (THERMO FISHER SCIENTIFIC, Illkirch, France). *EPHX2* genotyping consisted in the assessment of the presence of the common exonic SNPs of *EPHX2* K55R (rs415907953) R287Q (rs751141) and of the most prevalent intronic SNPs 3′UTR (rs1042032) and rs6558004. rs6558004 was in fact identified by a bioinformatic analysis and allows to study 19 intronic SNPs with a strong linkage disequilibrium (LD > 0.90) and a minor allele frequency higher than 15% (Supplementary Table 1)^24^. Similarly, the presence of LoF 2C8*3 (rs10509681 and rs11572080), 2C9*2 (rs1799853), 2C9*3 (rs1057910), 2C19*2 (rs4244285) and 2J2*7 (rs890293) SNPs and of the gain-of-function (GoF) CYP2C19*17 (rs12248560) polymorphism was assessed. All these CYP generate EETs from arachidonic acid and the presence of their SNPs were shown to be associated with some cardiovascular diseases^25,26^.

**Table 1:** Clinical characteristics of kidney transplant recipients.

Parameters	Subjects (n = 79)
Time after transplantation, years	5.4 ± 3.7
Age, years	54 ± 13
Male, n (%)	56 (71%)
Body mass index, kg/m^2^	27.2 ± 5.2
3-month eGFR, ml/min/1,73 m^2^	54 ± 21
6-month eGFR, ml/min/1,73 m^2^	52 ± 19
12-month eGFR, ml/min/1,73 m^2^	52 ± 19
eGFR at exploration visit, ml/min/1,73 m^2^	52 ± 22
Donor age, years	48 ± 14
Cold ischemia time, hours	15 ± 7
**Immunosuppressive agent, n (%)**	
Cyclosporine	18 (23%)
Tacrolimus	59 (75%)
Mycophenolate Mofetil	70 (89%)
Hypertension, n (%)	73 (92%)
Diabetes, n (%)	16 (20%)
Statins, n (%)	49 (62%)

Data are mean ± SD or n (%).eGFR: estimated glomerular filtration rate.

In addition, peripheral blood mononuclear cells (PBMCs) were isolated from whole blood by density gradient centrifugation for further determination of sEH protein activity using a [^3^H]-trans-1,3-diphenylpropene oxide-based radioactivity assay^23,27^. The sEH activity was normalized to total protein level measured by the BCA method.

Blood sampling at 34 and 44 °C allowed the quantification of plasma 8,9-EET, 11,12-EET, 14,15-EET and corresponding DHET regioisomers using a recently validated method^22^. Because more than 99% circulate bound to lipoproteins, total EETs were determined after plasma lipid extraction and saponification by liquid chromatography coupled to tandem mass spectrometry analysis^22,23^. The variations in total EETs + DHETs levels between 34 and 44 °C was used as an index of EET production during the endothelial stimulation with heating^22,23^.

### Impact of sEH inhibition in a murine model of renal ischemia reperfusion injury

#### Animals and treatment

The protocol was approved by a local institutional review committee (Comité d'Ethique NOrmandie en Matière d'EXpérimentation Animale—CENOMEXA, no. 01353.03) and conducted in accordance with the National Institutes of Health (NIH) Guide for the Care and Use of Laboratory Animals and the ARRIVE guidelines (http://www.nc3rs.org.uk/page.asp?id=1357). All experiments were carried out in 129/Sv male mice, aged 8 weeks and weighing between 20 and 26 g (January laboratory, Genest Isle). The surgical procedures were performed by a single experienced operator in order to ensure reproducibility. Briefly, bilateral I/R was induced in anesthetized mice (10 mg/kg xylazine, 100 mg/kg ketamine) by a 30-min clamping of renal vascular pedicles as previously described^28^. Immediately after the surgical procedure, I/R mice were randomized into two groups to receive either the sEH inhibitor *trans*-4-(4-(3-adamantan-1-yl-ureido)-benzoic acid (*t*-AUCB: 10 mg/l in drinking water after dilution in 10-mL PEG 400) or vehicle (PEG 400) until sacrifice^29^. A third group of sham-operated mice (surgical laparotomy) served as controls.

#### Renal parameters

Sacrifices were performed 4 weeks after surgery. Blood samples were collected from the aorta in anesthetized animals (10 mg/kg xylazine, 100 mg/kg ketamine), allowing to measure plasma creatinine.

Kidneys were removed, weighed and histological lesions were analysed after Masson's staining as previously described^30^. The slides were independently examined on a blinded basis, using a 0 to 4 injury scale for the level of interstitial inflammation, interstitial fibrosis and glomerulosclerosis at magnification × 20 (0: no damage; 1: < 25% of kidney damaged; 2: 25–50% of kidney damaged; 3: 50–75% of kidney damaged; 4: 75–100% of kidney damaged). Tubular lesions were analysed at magnification × 10. Vascular thickening and vascular fibrosis were analysed at magnification × 40.

#### Cardiovascular parameters

In mice anesthetized with isoflurane (1 to 2%), left ventricular (LV) dimensions and function were assessed two days before the sacrifice, using a Vivid 7 ultrasound device (GE MEDICAL)^29^. The heart was imaged in the 2D mode in the parasternal short axis view. With the use of M mode image, LV end-diastolic (EDD) and systolic diameters (ESD) were measured. Ejection fraction (EF) was calculated from the LV cross-sectional area as EF (%) = ((LVDA-LVSA)/LVDA) × 100 where LVDA and LVSA are LV diastolic and systolic area, estimated from EDD and ESD. Furthermore, Doppler measurements were made at the tip of the mitral leaflets for diastolic filling profiles in the apical four-chamber view, allowing to determine peak early (E) and late (A) mitral inflow velocities, and calculation of the E/A ratio, as index of LV diastolic function.

Renal artery vascular reactivity was evaluated 4 weeks after surgery by myography (Dual Wire Myograph System, DANISH MYO TECHNOLOGY). For this purpose, the distal segment of right renal artery, 1 mm-long and ~ 250 µm in diameter, was carefully dissected and mounted in a small vessel myograph for isometric tension recording. All measurements were performed after vessel contraction with 10^–6^ M phenylephrine. The endothelium-dependent relaxations to acetylcholine (10^–9^–10^–4.5^ M) and endothelium-independent relaxations to sodium nitroprusside (10^–9^–10^–4.5^ M) were assessed^31^.

Finally, the heart was harvested, weighed and a section of the left ventricle was snap frozen for subsequent determination of LV collagen density, using 8-µm-thick histological slices stained with Sirius Red^31^.

### Statistical analysis

Continuous variables are presented as mean ± standard deviation (SD) unless differentially indicated. Categorical variables are presented as frequency and percentage. Statistical analysis was performed using R software (version 3.4.4) and NCSS software (version 07.1.14).

For the human genetic study, Hardy–Weinberg Equilibrium for the 4 tag-SNPs of *EPHX2* and for *CYP450* SNPs were assessed using exact test from the *genetics* package for R^32^. Correlations between *EPHX2* and *CYP450* SNPs were assessed using the *genetics* package for R^30^. The r correlation coefficient was used using the following formula: r = − D/sqrt(p(A) * p(a) * p(B) * p(b)) with D, corresponding to the raw difference in frequency between the observed number of AB pairs and the expected number as follow: D = p(AB) − p(A)*p(B) where—p(A) is defined as the observed probability of allele ‘A’ for marker 1, − p(a) = 1 − p(A) is defined as the observed probability of allele ‘a’ for marker 1, − p(B) is defined as the observed probability of allele ‘B’ for marker 2, − p(b) = 1 − p(B) is defined as the observed probability of allele ‘b’ for marker 2, and − p(AB) is defined as the probability of the marker allele pair ‘AB’.

Predictors of eGFR at 3, 6 and 12 months and of eGFR and vascular function at the time of exploration were assessed using *leaps* package for R^33^. This package uses a branch-and-bound algorithm to find the best predictive model according to adjusted R-squared performing an exhaustive search for the best subset of variables^34^. The following variables have been included for predictors of eGFR at 3, 6 and 12 months: sex, BMI, cyclosporine use (0 = no, 1 = yes), donor age, recipient age at the time of transplantation, cold ischemia time, the presence of *EPHX2* and *CYP450* SNPs either as heterozygous and homozygous alleles (= MT). Recipient age at the time of the exploration visit was added as covariable for analysis of eGFR and vascular parameters determined that day. Once the predictors retained from leaps results, a parametric bootstrap with 1000 replications was performed with *boot* package for R^35^, allowing to approximate the distribution of the predictor coefficients.

In addition, based on SNPs identified by leaps analysis and associations between them, carrier and non-carrier patients were compared either directly or using haplotype constructions. Thus, each single SNP or haplotype was compared with non-mutated wild-type (WT) alleles, using multiple linear regression with all other predictors identified in leaps analysis, and WT was considered as the reference level. This last approach was also performed to assess the impact of these SNPs on sEH activity, EET and DHET basal bioavailability and variation in EETS + DHETs during hand skin heating.

For the experimental study, sham-operated, vehicle-treated and *t*-AUCB-treated renal I/R groups were compared using ANOVA followed by Tukey multiple comparison post-tests.

A *P* value < 0.05 was considered statistically significant.

## Results

### Human genetic study

Exploration visits were performed 5.4 ± 3.7 [min–max: 1.1–16.1] years after kidney transplantation. Clinical characteristics of patients are summarized in Table 1.

#### Genotyping

**Figure 1:**
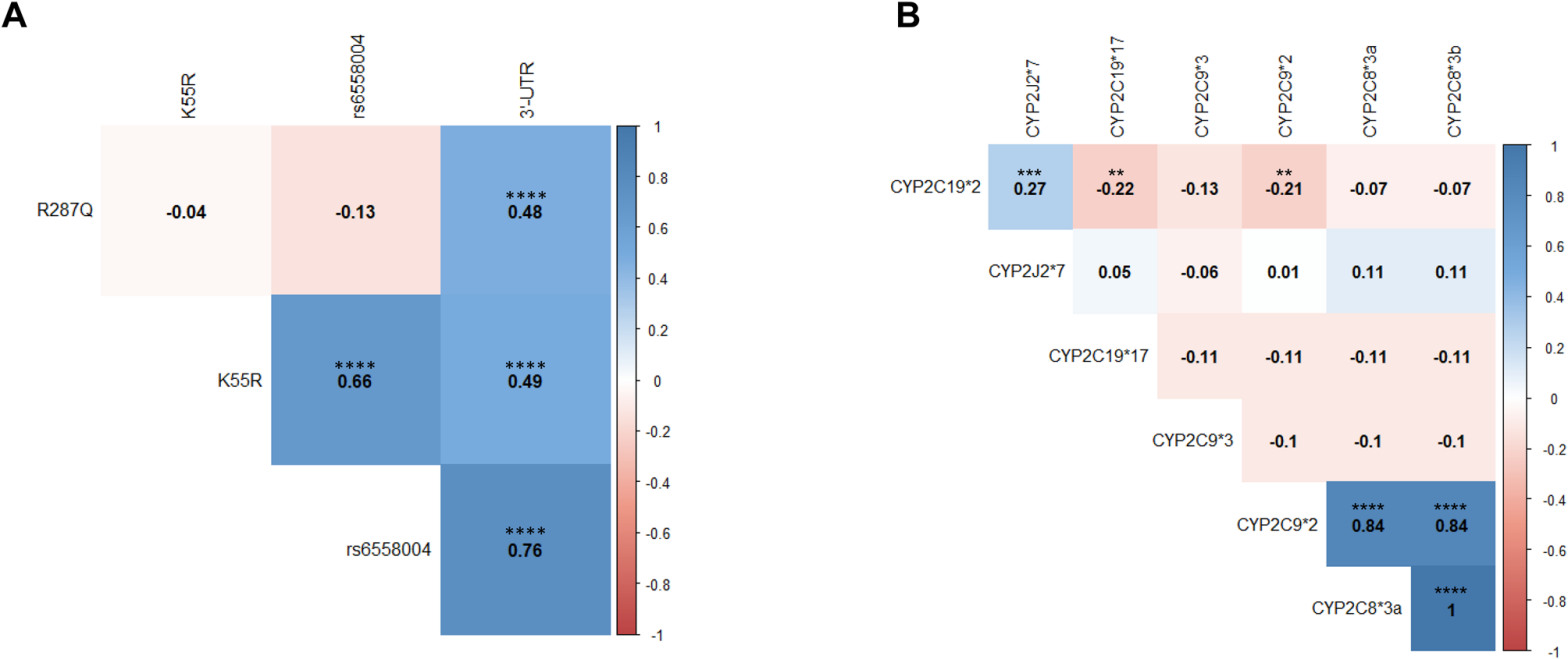
Correlation between the SNPs of *EPHX2* (**A**) and *CYP450* (**B**) determined in kidney transplant recipients. Pairwise linkage disequilibrium was expressed as correlation coefficient (r) determined using the following formula: r =  *− *D/sqrt(p(A) * p(a) * p(B) * p(b)) with D, corresponding to the raw difference in frequency between the observed number of AB pairs and the expected number as follow: D = p(AB) − p(A)*p(B). *P* value is the Chi-square *p* value for marker independence.

The frequency of each genotype for the *EPHX2* and *CYP450* SNPs was in agreement with Hardy–Weinberg equilibrium based on the MAF from the HapMap-CEU population listed at https://www.ncbi.nlm.nih.gov/snp/ (Supplemental Table 2). Interestingly, strong associations between several *EPHX2* SNPs were observed (Fig. 1A and Supplemental Table 3). In particular, the 3′-UTR SNP was always associated with R287Q or with a less studied intronic SNP rs6558004 (or with both rs6558004 and K55R polymorphisms). In addition, CYP2C8*2 and CYP2C8*3 SNPs were always associated and most of the time with CYP2C9*2 (Fig. 1B and Supplemental Table 3).

**Table 2:** Multiple linear regression of estimated glomerular filtration rate (eGFR) according to the predictors obtained from leaps analysis.

Parameters	Predictors	Estimate (± SE)	T	*P* value
3-month eGFR	Intercept	112.0 ± 11.7	10.03	< 0.001
	Donor age	− 0.8 ± 0.1	− 5.96	< 0.001
	Body mass index	− 0.5 ± 0.4	− 1.27	0.21
	Cyclosporine use	− 6.7 ± 4.5	− 1.51	0.14
	Cold ischemia time	− 0.6 ± 0.3	− 2.11	0.039
	3′-UTR = MT	− 10.0 ± 5.7	− 1.74	0.086
	rs6558004 = MT	16.8 ± 5.8	2.91	0.005
6-month eGFR	Intercept	112.8 ± 8.8	12.83	< 0.001
	Donor age	− 0.8 ± 0.1	− 7.60	< 0.001
	Body mass index	− 0.6 ± 0.3	− 1.83	0.071
	Cyclosporine use	− 8.7 ± 3.7	− 2.38	0.020
	Cold ischemia time	− 0.5 ± 0.2	− 2.13	0.036
	rs6558004 = MT	5.6 ± 3.1	1.79	0.077
12-month eGFR	Intercept	115.7 ± 8.0	14.46	< 0.001
	Donor age	− 0.9 ± 0.1	− 9.00	< 0.001
	Body mass index	− 0.7 ± 0.3	− 2.35	0.022
	Cyclosporine use	− 7.7 ± 3.6	− 2.15	0.035
	Cold ischemia time	− 0.4 ± 0.2	− 1.80	0.076
	rs6558004 = MT	6.4 ± 3.9	1.63	0.11
	K55R = MT	− 7.9 ± 4.9	1.60	0.11
	2C19*2 = MT	3.7 ± 2.9	1.31	0.20
eGFR at exploration visit	Intercept	121.0 ± 11.1	10.92	< 0.001
	Donor age	− 0.9 ± 0.1	− 7.15	< 0.001
	Body mass index	− 0.9 ± 0.4	− 2.60	0.011
	Cyclosporine use	− 12.5 ± 4.5	− 2.76	0.008
	3′-UTR = MT	− 19.5 ± 12.5	− 1.56	0.12
	rs6558004 = MT	35.1 ± 13.2	2.66	0.010
	K55R = MT	− 17.0 ± 6.6	− 2.56	0.013
	R287Q = MT	12.9 ± 12.3	1.05	0.30
	2C9*3 = MT	13.5 ± 5.8	2.33	0.023

**Table 3:** Multiple linear regression of radial artery vascular function according to the predictors obtained from leaps analysis.

Parameters	Predictors	Estimate (± SD)	T	*P* value
Flow-mediated dilatation	Intercept	19.1 ± 5.4	3.54	< 0.001
	Recipient age	− 0.2 ± 0.05	− 4.33	< 0.001
	Body mass index	− 0.2 ± 0.1	− 1.59	0.12
	Male = MT	− 7.5 ± 1.5	− 5.10	< 0.001
	R287Q = MT	3.9 ± 1.8	2.14	0.036
	2C8*3 = MT	5.6 ± 2.5	2.24	0.028
	2C9*2 = MT	− 5.8 ± 2.5	− 2.34	0.022
	2C9*3 = MT	− 3.6 ± 2.1	− 1.72	0.090
	2C19*17 = MT	− 2.2 ± 1.5	− 1.48	0.15
GTN-induced dilatation	Intercept	23.5 ± 5.6	4.23	< 0.001
	Body mass index	− 0.4 ± 0.15	− 2.69	0.009
	Male = 1	1.9 ± 1.8	1.06	0.29
	Cyclosporine use	− 2.4 ± 1.8	− 1.30	0.20
	Cold ischemia time	0.2 ± 0.1	1.52	0.13
	R287Q = MT	4.8 ± 2.2	2.20	0.032
	2C9*3 = MT	− 2.6 ± 2.5	− 1.05	0.30
	2C19*17 = MT	4.1 ± 1.8	2.33	0.023
	2J2*7 = MT	− 3.0 ± 2.5	− 1.24	0.22

#### Impact of genetic variations of EPHX2 and CYP450 on renal function

Based on the best subset of variables provided from the branch-and-bound algorithm (Supplemental Fig. 1A–D), Leaps analysis revealed that the main predictors of short term eGFR measured up to 12 months after transplantation are donor age, cold ischemia time, BMI, cyclosporine use and presence in the recipient of the *EPHX2* SNPs 3′-UTR, K55R and/or rs6558004 (full model: R^2^ = 0.43 at 3 months, R^2^ = 0.54 at 6 months and R^2^ = 0.61 at 12 months, Table 2). A long-term protective effect on renal allograft function associated with rs6558004 and a deleterious effect with K55R was clearly demonstrated at the time of the exploration visit (R^2^ = 0.51), while their short-term impact appears less marked (Table 2). None of the *CYP450* SNPs was identified as a predictive variable of eGFR evaluated at these different time points apart from the CYP2C9*3 SNP that was unexpectedly associated with protection against allograft dysfunction at the time of the exploration visit (Table 2).

**Figure 2:**
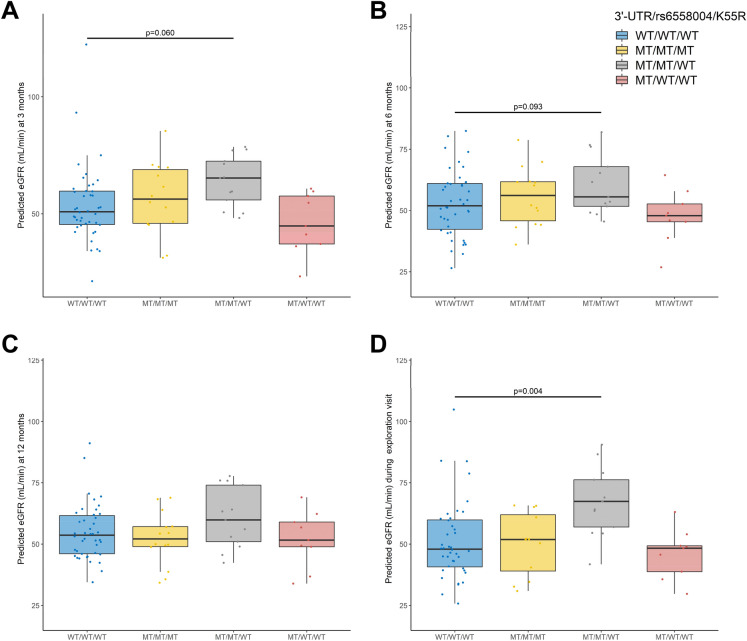
Impact of the haplotype construction of 3′UTR, rs65598004 and K55R polymorphisms on predicted estimated glomerular filtration rate (eGFR) adjusted to other covariates determined 3 (**A**), 6 (**B**) and 12 (**C**) months after transplantation, and at the time of the exploration visit (**D**).

The impact of the 3 *EPHX2* SNPs identified by Leaps analysis on renal function was further explored taking into consideration their previously described associations using the following haplotypes: WT/WT/WT, 3′UTR/rs6558004/K55R, 3′UTR/rs6558004/WT and 3′-UTR/WT/WT. This approach confirmed the long-term protective effect of rs6558004 on renal function, a protective effect that is lost in patients also carrying K55R (Figs. 2A–D). In addition, the comparison of carriers and non-carriers of CYP2C9*3 confirmed the protective effect of this SNP at the time of the exploration visit (Supplemental Fig. 2).

#### Impact of genetic variations of EPHX2 and CYP450 SNPs on vascular function

**Figure 3:**
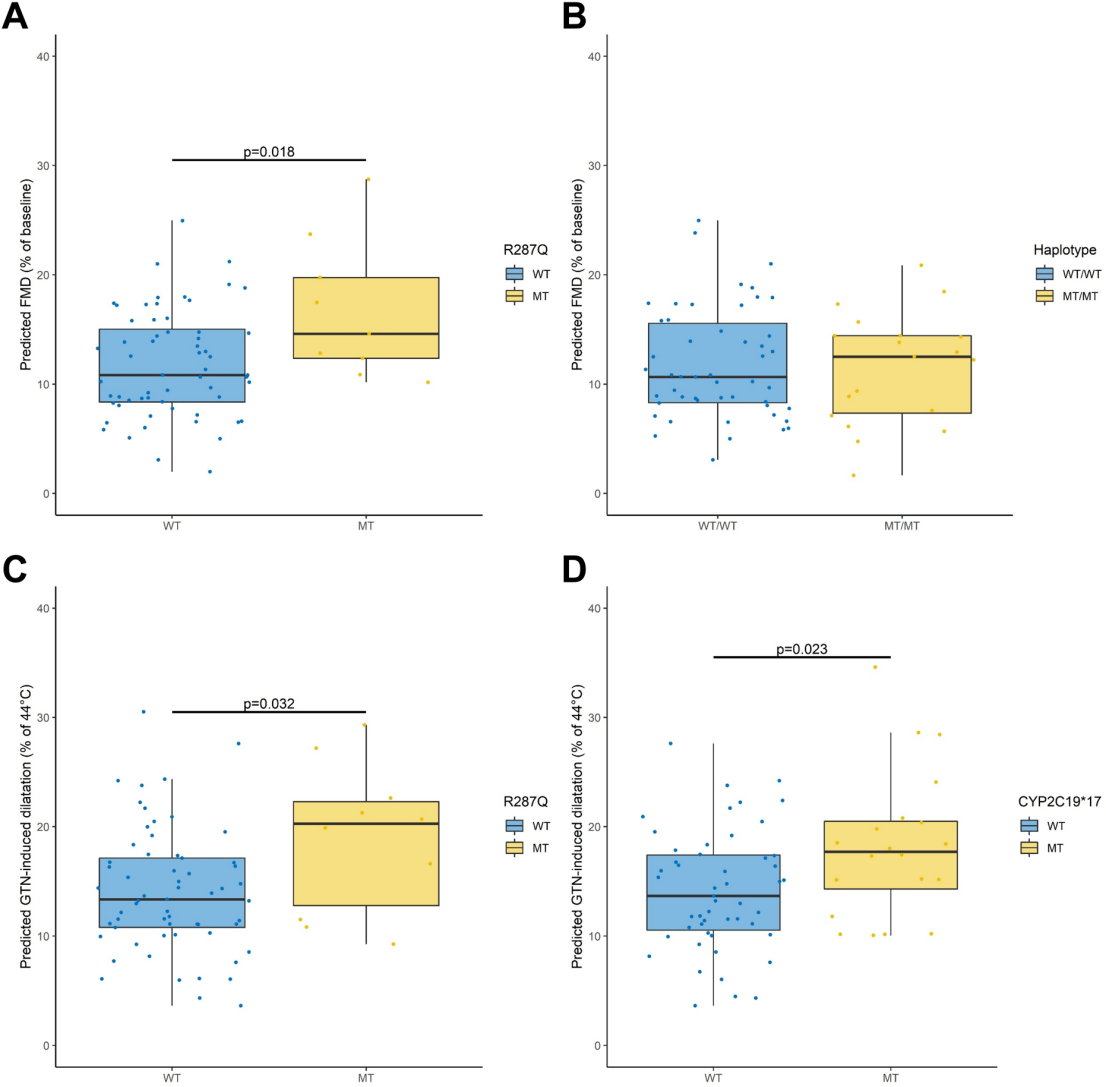
Impact of R287Q (**A**) and CYP2C8*3/2C9*2 (**B**) polymorphisms on radial artery endothelium-dependent flow-mediated dilatation in response to hand skin heating and of R287Q (**C**) and CYP2C9*17 (**D**) on endothelium-independent dilatation to glyceryl trinitrate (GTN) adjusted to other covariates determined at the time of the exploration visit in kidney transplant recipients.

Based on the best subset of variables provided from the branch-and-bound algorithm (Supplemental Fig. 3A), Leaps analysis revealed that the main predictors of the magnitude of endothelium-dependent flow-mediated dilatation in response to heating are recipient age, sex, the presence of the R287Q, CYP2C8*3 and CYP2C9*2 genotypes (R^2^ = 0.44; Table 3). Comparisons of carriers and non-carriers tended to confirm the beneficial impact of R287Q on flow-mediated dilatation (Fig. 3A). In contrast, the collinearity between CYP2C8*3 and 2C9*2 did not allow to evaluate their opposite impact on endothelial function. The comparison of patients carrying these genotypes to non-carriers yielded no difference in flow-mediated dilatation (Fig. 3B).

Based on the best subset of variables provided from the branch-and-bound algorithm (Supplemental Fig. 3B), Leaps analysis revealed that the main predictors of the magnitude of endothelium-independent dilatation in response to glyceryl trinitrate are BMI and the presence of R287Q and CYP2C19*17 genotypes (R^2^ = 0.17; Table 3). Comparisons of carriers and noncarriers confirmed the positive impact of both genotypes on endothelium-independent dilatation (Fig. 3C,D).

#### Impact of EPHX2 and CYP450 SNPs identified as modifying factors of renal and vascular function on EET pathway

**Figure 4:**
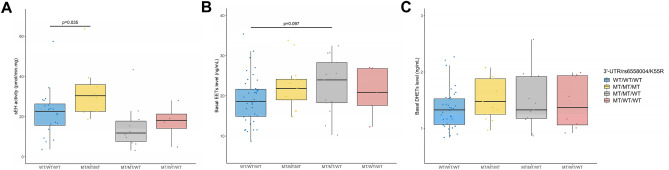
Impact of the haplotype construction of 3′UTR, rs65598004 and K55R polymorphisms on soluble epoxide hydrolase (sEH) activity in isolated peripheral blood mononuclear cells (**A**), baseline plasma levels of epoxyeicosatrienoic acids (EETs; **B**) and dihydroxyeicosatrienoic acids (DHETs; **C**) determined at the time of the exploration visit in kidney transplant recipients.

**Figure 5:**
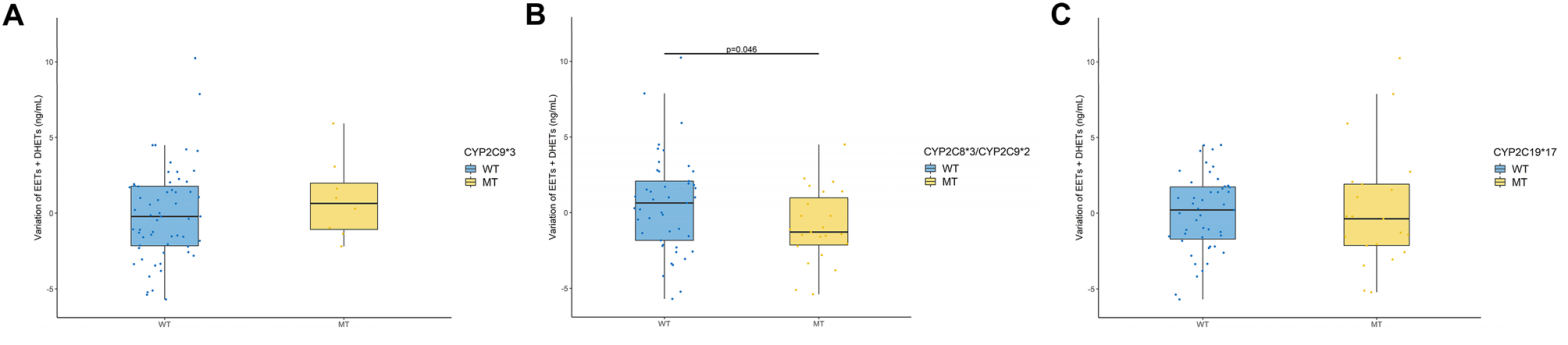
Impact of CYP2C9*3 (**A**), CYP2C8*3/2C9*2 (**B**) and CYP2C19*17 (**C**) polymorphisms on the variations of plasma levels of epoxyeicosatrienoic acids and dihydroxyeicosatrienoic acids (EETs + DHETs).

Compared to the most common genotype, the sEH activity was increased in K55R carriers but was not significantly modified by rs6558004 (Fig. 4A). K55R did not affect EET baseline levels while rs6558004 tended to increase them (Fig. 4B), without affecting DHET levels (Fig. 4C), this effect being in particular related to an elevation of 8,9-EET regio-isoform (Supplemental Fig. 4). None of these parameters were affected by R287Q (Supplemental Fig. 5). Carriers of CYP2C9*3, CYP2C8*3/2C9*2 or CYP2C19*17 genotypes had similar baseline levels of EETs and DHETs (Supplemental Fig. 6) and only carriers of CYP2C8*3 and CYP2C9*2 polymorphisms had a reduced variation in EETs + DHETs during the endothelial stimulation with heating (Fig. 5).

**Figure 6:**
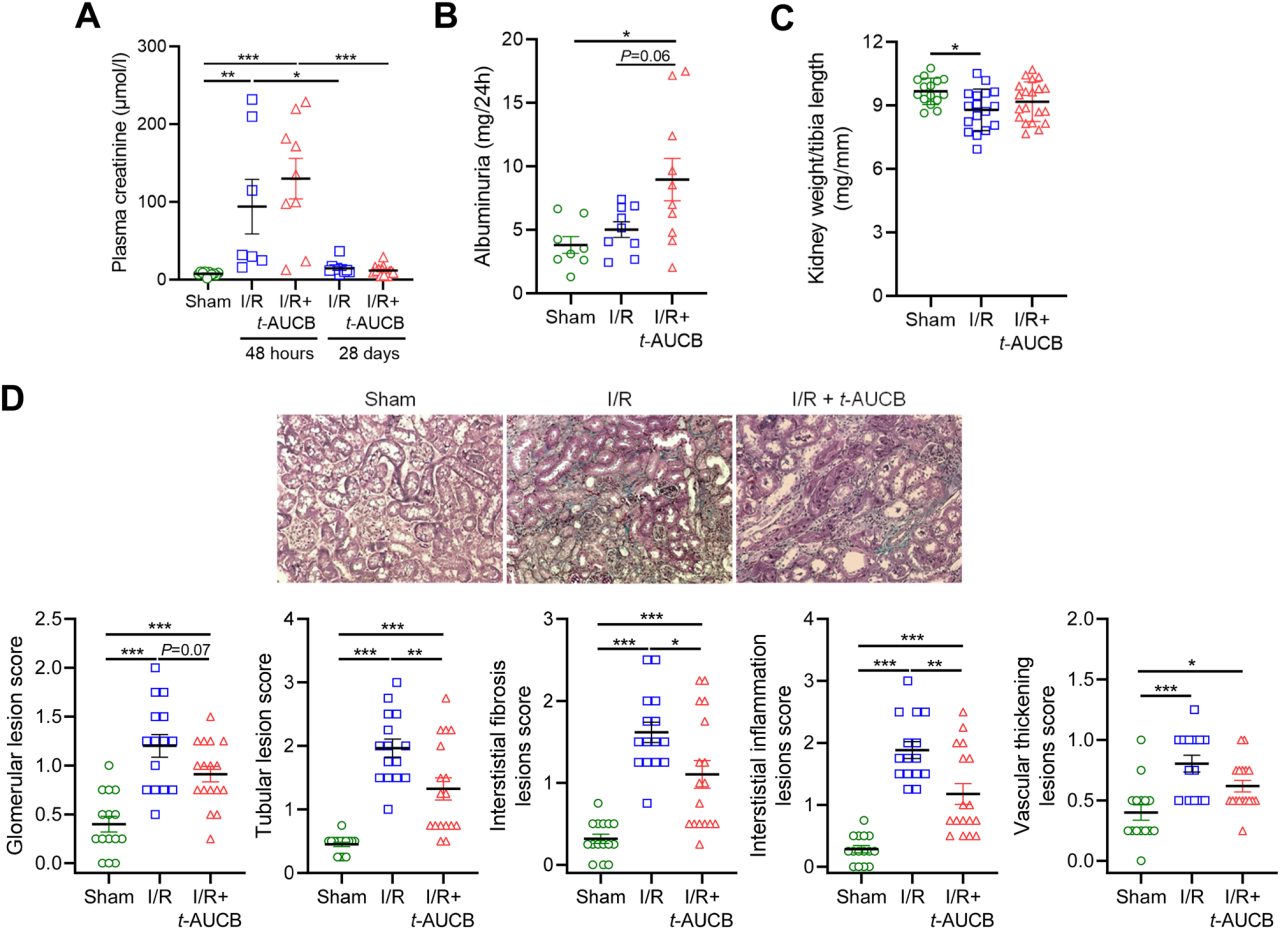
Impact of soluble epoxide hydrolase inhibition on kidney damage induced by renal-ischemia reperfusion injury. Plasma creatinine at day 2 and day 28 after surgery (**A**), 24-h urine albumin excretion (**B**), kidney weight (**C**), representative images and scoring of kidney lesions at sacrifice (**D**) 28 days after surgery in sham-operated mice and mice subjected to bilateral renal ischemia/reperfusion (I/R) treated or not with the soluble epoxide hydrolase inhibitor *t*-AUCB. *P < 0.05.

### Animal study

#### Renal parameters

Plasma creatinine markedly increased 48 h after I/R in mice treated with vehicle or with *t*-AUCB, compared to sham-operated mice, and returned to near baseline levels 28 days after surgery without difference between groups (Fig. 6A). Twenty-four-hour urine albumin excretion increased significantly in I/R mice treated with *t*-AUCB but not in vehicle-treated I/R mice 28 days after surgery (Fig. 6B). This was observed while 28-day treatment with *t*-AUCB had no impact in sham-operated mice (sham: 3.83 ± 0.66 vs. sham + *t*-AUCB: 3.67 ± 1.57 mg/24 h, *P* = 0.35). In contrast, kidney weight was reduced in I/R mice treated with vehicle while this reduction was not significant in I/R mice treated with *t*-AUCB (Fig. 6C). Histological analyses revealed glomerulosclerosis, tubular injury, interstitial inflammation and fibrosis, and vascular thickening in both groups of I/R mice but all lesions were significantly less marked in mice treated with *t*-AUCB (Fig. 6D).

#### Cardiovascular parameters

**Figure 7:**
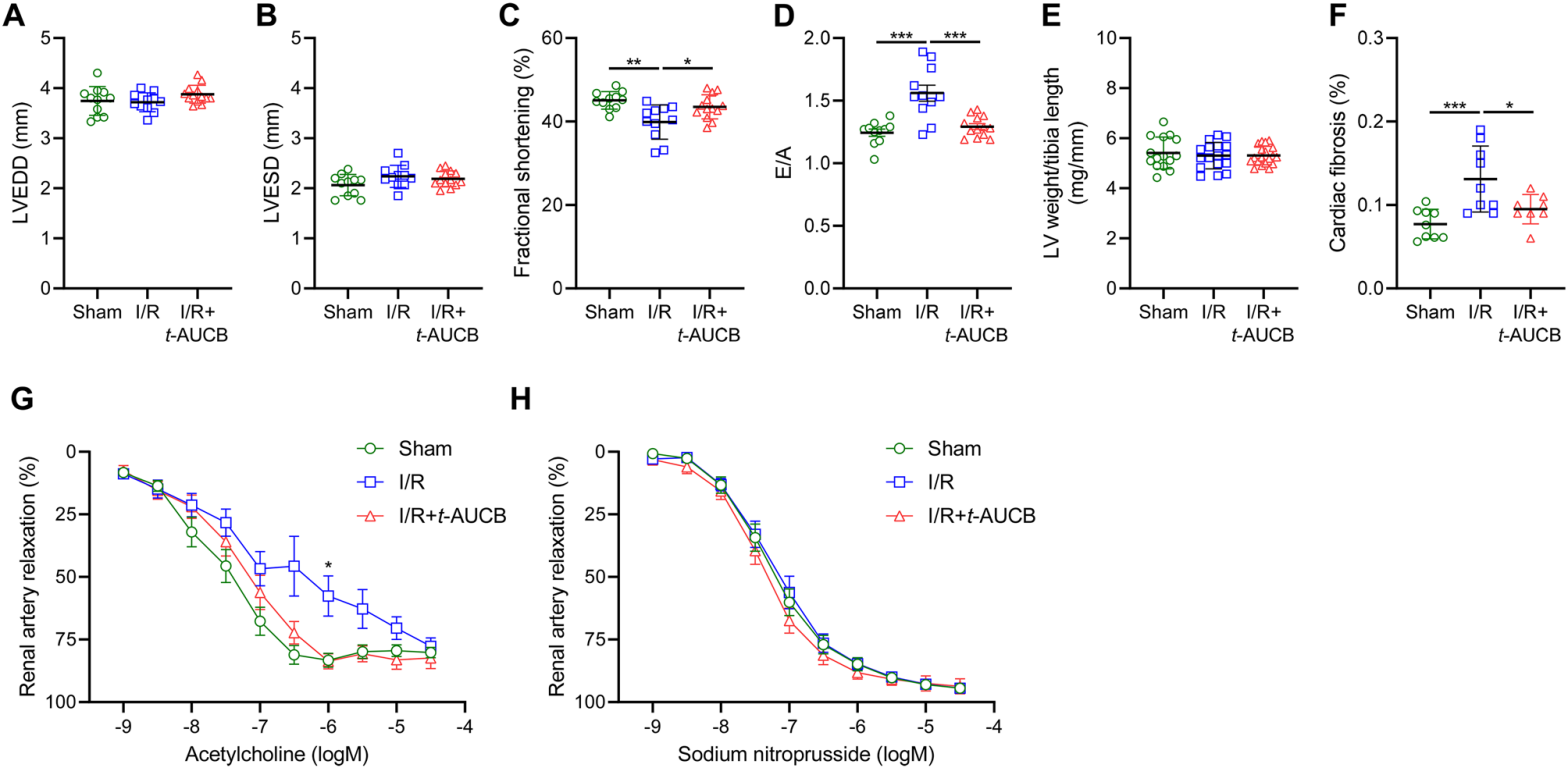
Impact of soluble epoxide hydrolase inhibition on cardiovascular alterations induced by renal-ischemia reperfusion injury Left ventricular end-diastolic (LVEDD; **A**) and end-systolic (LVESD; **B**) diameters, fractional shortening (**C**), E/A ratio (**D**), LV weight (**E**), cardiac fibrosis (**F**), endothelium-dependent relaxations to acetylcholine (**G**) and endothelium-independent relaxations to sodium nitroprusside (**H**) determined 28 days after surgery in sham-operated mice and mice subjected to bilateral renal ischemia/reperfusion (I/R) treated or not with the soluble epoxide hydrolase inhibitor *t*-AUCB. *P < 0.05.

Echocardiography showed no significant change in LV end-diastolic and systolic diameters but a decrease in fractional shortening and E/A ratio in I/R mice treated with the vehicle compared to sham-operated mice (Fig. 7A–D). The decreases in fractional shortening and E/A ratio were prevented by *t*-AUCB, without change in LV diameters. Heart weight was not significantly different between groups (Fig. 7E). Cardiac fibrosis was increased in I/R mice receiving the vehicle and this increase was alleviated in I/R mice receiving *t*-AUCB (Fig. 7F).

The potency of acetylcholine to induce renal artery endothelium-dependent relaxation was slightly reduced in I/R mice receiving the vehicle but not in I/R mice treated with *t*-AUCB compared to sham-operated animals, without difference between groups for endothelium-independent relaxation to sodium nitroprusside (Fig. 7G,H).

## Discussion

The main finding of the present work is that in kidney transplant recipients the presence of genetic variants of *EPHX2* and *CYP450* differentially modulate short- and long-term allograft function and vascular function, depending on their effect on sEH activity and/or EET bioavailability. Overall, the genetic data supports the hypothesis that reduction of sEH activity and increase in EET bioavailability are protective in this context. Moreover, a beneficial effect of a pharmacological decrease in sEH activity on renal and cardiovascular homeostasis was directly confirmed in a murine model mimicking the I/R injury suffered by the transplanted kidney.

To assess whether the modulation of EET pathway represents a promising therapeutic target to prevent the development of chronic allograft dysfunction and/or cardiovascular complications in kidney transplant recipients, a comprehensive genetic polymorphism study was first performed. The presence of the main SNPs of the EET producing and degrading enzymes was determined in a population of patients transplanted since at least one year and their impact on eGFR and vascular function were studied.

First of all, for the sEH, the Leaps analysis and haplotype construction both showed that, in addition to the classical predictors of eGFR, the presence of rs6558004 is associated with a higher eGFR and this impact appeared in particular more marked at the time of the exploration visit up to 16 years after transplantation. Interestingly, haplotype construction did not show a decreased sEH activity in carriers of the rs6558004 intronic mutation but these patients had higher plasma level of 8,9-EET, which was identified as the only EET regioisomer with a protective effect on the glomerular filtration barrier^36^. Although suggested to play little role for exonic *EPHX2* SNPs^37^, it could be speculated that this SNP and/or other of their associated intronic SNPs promotes variations in sEH structure and substrate selectivity that cannot be detected with the sEH activity assay used. Moreover, the Leaps analysis revealed that the presence of the K55R SNP is associated with a lesser eGFR at the time of the exploration visit, and haplotype constructions showed that patients carrying simultaneously K55R with rs6558004 lose the renal protective effect of the latter. As expected from in vitro studies with recombinant sEH^38^, patients carrying K55R had increased sEH activity. Plasma EETs and/or DHETs were not modified but the presence of K55R prevented the increase in 8,9-EET induced by rs6558004. Circulating levels may not reflect tissue concentration as K55R was predicted to induce small changes in the metabolism of epoxyfatty acids^37^. In addition, we cannot exclude that baseline EET level was already reduced in kidney transplant recipients compared to healthy populations, leading to underestimate the negative impact of the gain-of-function K55R. In fact, previous clinical studies only evaluated and showed the decrease in the ratio of epoxyoctadecenoic acid-to-dihydroxyoctadecenoic acid (EpOME-to-DiHOME), which are also produced by the CYP450-sEH pathway but from linoleic acid, in K55R carriers^16,39^. This was observed in some populations but not all^16,39^, suggesting that other factors besides sEH catalytic activity play a role in the diseases associated with this SNP^37^. None of the analyses clearly identified the 3′-UTR SNP as a predictor of eGFR although a trend for a poorer renal function was observed in the Leaps analysis 3 months post-transplantation. This result could be explained either by the absence of impact of the 3′-UTR or by its association with rs6558004 or with R287Q, which may be also protective, thus masking its possible deleterious effect. In a previous study, haplotype construction, including R287Q and K55R, also showed a trend for a deleterious impact of the 3′-UTR SNP on the outcome of deceased-donor renal transplantation in Caucasians^15^. To note, the limited number of subjects included in the present study may have lea to only detect the greatest effects of *EPHX2* SNPs on renal function.

Furthermore, none of the *CYP450* SNPs assessed affected allograft function in the short term and only one, CYP2C9*3, appeared to positively affect allograft function in the long term. This unexpected finding is observed while no apparent modifications in EETs or DHETs are observed. We cannot exclude that the presence of this SNP in only 8 patients may have led to a misleading type II error. However, hypothetically, this LoF polymorphism may reduce the bioavailability of other epoxyfatty acids derived from omega-3 that are known to be efficiently produced by CYP2C9 and have recently been shown to potentiate experimental kidney injury^34,40^. The weak impact of *CYP450* polymorphisms on allograft function was also reported in one study showing that only CYP2J2*7 SNP of the donor but not of the recipient negatively affects the outcome of renal transplantation^18^.

At the vascular level, the presence of CYP2C8*3 and CYP2C9*2 SNPs seemed to exert opposite effects on endothelium-dependent flow-mediated dilatation but the strong associations between both precludes the direct analysis of their real impact. However, carriers of these LoF polymorphisms had no change in EET or DHET basal bioavailability but displayed a reduced variation in EET + DHET during the sustained endothelial stimulation induced by heating. Given the major role of EETs in sustained flow-mediated dilatation^21–23^, the reduced EET production in response to the increase in blood flow may promote endothelial dysfunction of conduit arteries in kidney transplant recipients. This mechanism may contribute to the deleterious impact of LoF *CYP450* SNPs on cardiovascular outcomes in this population^41^. Furthermore, patients carrying R287Q had a higher magnitude of flow-mediated dilatation but also of endothelium-independent dilatation to GTN. This surprising result had been previously observed in one study evaluating the impact of *EPHX2* polymorphisms at the arteriolar level^42^. Indeed, endothelium-independent dilatation of forearm resistance arteries in response to local infusion of sodium nitroprusside was enhanced in R287Q carriers and the authors speculated that this effect was related to a long-term preventive effect of increasing EET bioavailability against vascular remodelling^42^. In support of this hypothesis, carriers of the only GoF SNP CYP2C19*17 also had increased endothelium-dependent dilatation. Modification in sEH activity was not detected in carriers of R287Q but the low number of subjects and the association of this SNP with K55R in some patients may explain this result. In addition, no change in EET and DHET levels was observed in R287Q and CYP2C19*17 carriers. As previously discussed for K55R, the absence of modifications in circulating EETs or DHETs bioavailability may not reflect tissue levels and, in R287Q carriers, only the circulating EpOME-to-DiHOME ratio but not EET-to-DHET ratio was increased as expected^43,44^.

Then, the interest of inhibiting sEH in this setting was further investigated with a pharmacological approach in mice subjected to kidney I/R. Indeed, I/R is a major determinant of short and long-term allograft dysfunction in kidney transplant recipients, in addition to allo-immune and nephrotoxic mechanisms^45^. As expected, plasma creatinine dramatically increased shortly after I/R and largely improved with time. However, plasma creatinine did not fully recover and kidney lesions were present at sacrifice, reflecting the particular severity of the model. This is notably due to the genetic background used, which is known to be particularly prone to kidney injury^46,47^, the duration of ischemia that was longer than generally performed in mice^28^ and the global clamping of renal vascular pedicle, with both artery and vein^28^. In accordance with the severity of this model, endothelial vasomotor alterations, LV fibrosis and cardiac diastolic and systolic dysfunction developed, as previously demonstrated in subtotal nephrectomy^46^.

Interestingly, kidney lesions, especially interstitial inflammation, were clearly reduced at day 28 after I/R in mice treated with the sEH inhibitor *t*-AUCB. This model of I/R is characterized by rapid recovery of kidney function, and the transient increase in creatinine was not significantly affected by *t*-AUCB. These results suggest that the pharmacological blockade of sEH may be more effective in preventing the deleterious mechanisms associated with renal I/R that contribute to allograft dysfunction, especially inflammation, than those involved in the development of chronic kidney disease. As recently reviewed^48^, the renoprotective effects of sEH inhibition appear mostly believed to be ascribed to the increased renal levels of EETs with respect to their potent anti-inflammatory and antifibrotic actions. In the present work, the impact of *t*-AUCB may be related at least partly to the antiapoptotic effects of EETs that have been shown to protect tubular cells against I/R and its consequences through inhibition of NF-κB and MAPK activation and restoration of glycogen synthase kinase 3β (GSK3β) phosphorylation^39,40,48,49^. However, one should keep in mind that blockade of sEH may also exerts detrimental effect in this context by potentiating the bioavailability of epoxyfatty acids derived from omega-3 that, at the opposite, potentiates tubular cell apoptosis by worsening the reduction in GSK3β phosphorylation^40^. Furthermore, cardiovascular explorations showed for the first time that *t*-AUCB also prevented the cardiac dysfunction and remodelling as well as the renal endothelial dysfunction induced by I/R. Such beneficial effects are of particular interest in kidney recipients who are also more likely to experience cardiovascular complications than the general population. Of importance to define future human therapeutic strategy, it should be interesting to know whether this beneficial effect of sEH inhibition on renal inflammation and cardiovascular complications persists in the long-term in particular even after stopping drug administration.

Altogether, the indirect evidence from the human genetic association study and the results obtained in animal experiments support the preservation of EET bioavailability as a novel therapeutic option to prevent allograft dysfunction and cardiovascular complications in kidney transplant recipients, in particular through inhibition of sEH. A possible limitation of sEH inhibition that emerges in the context of kidney diseases is that the concomitant potentiation of other epoxyfatty acids with sEH inhibition, especially those derived from omega-3, may hamper the beneficial renal impact of EETs. More targeted strategies such as the use of EET analogues alone or combined with sEH inhibition, may further optimize the promising benefits of modulating CYP450-sEH pathway in kidney transplantation^39,49^.

## Supplementary Information

Supplementary Information 1.

## Abbreviations

3-UTR: *EPHX2* 3-Untranslated region
CYP450: Cytochrome P450
DHETs: Dihydroxyeicosatrienoic acids
E/A: Peak early (E) and late (A) mitral inflow velocities
EET: Epoxyeicosatrienoic acid
eGFR: Estimated glomerular filtration rate
GoF: Gain-of-function
I/R: Ischemia/reperfusion
LD: Linkage disequilibrium
LoF: Loss-of-function
LV: Left ventricular
MT: Homozygous alleles
PBMC: Peripheral blood mononuclear cell
sEH: Soluble epoxide hydrolase
SNP: Single nucleotide polymorphism
*t*-AUCB: *trans*-4-(4-(3-Adamantan-1-yl-ureido)-cyclohexyloxy)-benzoic acid
WT: Wild-type
