# Supplementary material for: Preservation of epoxyeicosatrienoic acid bioavailability prevents renal allograft dysfunction and cardiovascular alterations in kidney transplant recipients
Source: Sci Rep. 2021 Feb 12;11:3739. doi: 10.1038/s41598-021-83274-1 (PMC7881112; doi:10.1038/s41598-021-83274-1)

**Preservation of epoxyeicosatrienoic acid bioavailability prevents renal allograft dysfunction and cardiovascular alterations in kidney transplant recipients**

**Authors:** Thomas Duflot, Charlotte Laurent, Anne Soudey, Xavier Fonrose, Mouad Hamzaoui, Michèle Iacob, Dominique Bertrand, Julie Favre, Isabelle Etienne, Clothilde Roche, David Coquerel, Maëlle Le Besnerais, Safa Louhichi, Tracy Tarlet, Dongyang Li, Valéry Brunel, Christophe Morisseau, Vincent Richard, Robinson Joannidès, Françoise Stanke-Labesque, Fabien Lamoureux, Dominique Guerrot, Jérémy Bellien

**SUPPLEMENTS**

**SUPPLEMENTAL TABLES**

**Supplemental Table 1.** List of *EPHX2* single nucleotide polymorphisms (SNPs) detected by the *tag*-SNP rs6558004, rs41507953, rs751141 and rs1042032.

| **tag-SNP** | **MAF (%)** | **Detected SNP** | **Major/Minor allele** | **Position** | **Function** |
| --- | --- | --- | --- | --- | --- |
| **rs6558004** | 17.0 | rs6558004 | G / A | Chr 8 : 27535703 | Intron |
|  |  | rs7839733 | C / A | Chr 8 : 27507913 | Intron |
|  |  | rs7000388 | T / C | Chr 8 : 27514029 | Intron |
|  |  | rs891401 | C / G | Chr 8 : 27523677 | Intron |
|  |  | rs10503812 | G / A | Chr 8 : 27530682 | Intron |
|  |  | rs6558003 | G / A | Chr 8 : 27532675 | Intron |
|  |  | rs4149252 | G / T | Chr 8 : 27538594 | Intron |
|  |  | rs4149243 | T / C | Chr 8 : 27516406 | Intron |
|  |  | rs7843805 | G / A | Chr 8 : 27531940 | Intron |
|  |  | rs10283378 | T / C | Chr 8 : 27537732 | Intron |
|  |  | rs729609 | T / C | Chr 8 : 27539006 | Intron |
|  |  | rs10282967 | C / A | Chr 8 : 27537440 | Intron |
|  |  | rs6558002 | T / C | Chr 8 : 27532025 | Intron |
|  |  | rs4149248 | A / G | Chr 8 : 27527933 | Intron |
|  |  | rs7828349 | G / A | Chr 8 : 27535549 | Intron |
|  |  | rs7018249 | G / A | Chr 8 : 27514672 | Intron |
|  |  | rs4149239 | A / G | Chr 8 : 27505619 | Intron |
|  |  | rs4149245 | T / C | Chr 8 : 27521116 | Intron |
|  |  | rs4149247 | C / T | Chr 8 : 27527898 | Intron |
| **K55R** | 10.4 | rs41507953 | A / G | Chr 8 : 27500988 | K55R |
|  |  | rs17057284 | T / G | Chr 8 : 27508635 | Intron |
|  |  | rs13438899 | G / A | Chr 8 : 27537022 | Intron |
|  |  | rs13439459 | C / T | Chr 8 : 27536849 | S412S |
|  |  | rs7846038 | T / C | Chr 8 : 27504625 | Intron |
|  |  | rs17057288 | G / A | Chr 8 : 27510322 | Intron |
|  |  | rs41360445 | C / G | Chr 8 : 27509792 | Intron |
| **R287Q** | 9.7 | rs751141 | C / T | Chr 8 : 27516348 | R287Q |
|  |  | rs7357432 | A / C | Chr 8 : 27530328 | Intron |
| **3’-UTR** | 25.5 | rs1042032 | A / G | Chr 8 : 27544557 | 3' UTR |

All detected SNPs have a strong linkage disequilibrium (LD) higher than 0.90 and a minor allele frequency (MAF) higher than 1%.

**Supplemental Table 2.** Frequency of the *EPHX2* and *CYP450* SNP genotypes

| **SNP** | **Wild Type** | **Heterozygote** | **Homozygote** | **Calculated MAF (%)** | **Theoretic MAF (%)** | **Hardy-Weinberg equilibrium** |
| --- | --- | --- | --- | --- | --- | --- |
| rs6558004 | 51/79 | 26/79 | 2/79 | 19.0 | 17.0 | Yes (P=0.72) |
| K55R | 64/79 | 13/79 | 2/79 | 10.8 | 10.4 | Yes (P=0.21) |
| R287Q | 68/79 | 11/79 | 0/79 | 7.0 | 9.7 | Yes (P=1) |
| 3’-UTR | 43/79 | 33/79 | 3/79 | 24.7 | 25.5 | Yes (P=0.37) |
| CYP2C8*3 | 56/79 | 23/79 | 0/79 | 14.6 | 10.5 | Yes (P=0.35) |
| CYP2C9*2 | 56/79 | 23/79 | 0/79 | 14.6 | 11.9 | Yes (P=0.35) |
| CYP2C9*3 | 71/79 | 7/79 | 1/79 | 5.7 | 6.8 | Yes (P=0.21) |
| CYP2C19*2 | 50/79 | 25/79 | 4/79 | 20.9 | 14.9 | Yes (P=0.73) |
| CYP2C19*17 | 56/79 | 21/79 | 2/79 | 15.8 | 22.2 | Yes (P>0.99) |
| CYP2J2*7 | 69/79 | 10/79 | 0/79 | 6.3 | 6.4 | Yes (P>0.99) |

A P-value > 0.05 is consistent with Hardy-Weinberg Equilibrium assuming the null hypothesis that there is no significant difference between observed and expected genotypes.

**Supplemental Table 3.** Main haplotypes from *EPHX2* and *CYP450* SNPs (haplotype with n=1 has been removed).

| ***EPHX2* haplotypes**  **rs6558004/3’-UTR/K55R/R287Q** | **n=78/79** |
| --- | --- |
| WT/WT/WT/WT | 42 |
| **MT**/**MT**/WT/WT | 13 |
| **MT**/**MT**/**MT**/WT | 12 |
| WT/**MT**/WT/**MT** | 9 |
| **MT**/**MT**/**MT**/**MT** | 2 |
| ***CYP450* haplotypes**  **CYP2C8*3/CYP2C9*2/CYP2C9*3/CYP2C19*2/CYP2C19*17/CYP2J2*7** | **n=68/79** |
| WT/WT/WT/**MT**/WT/WT | 16 |
| WT/WT/WT/WT/WT/WT | 12 |
| WT/WT/WT/WT/**MT**/WT | 11 |
| **MT**/**MT**/WT/WT/WT/WT | 9 |
| **MT**/**MT**/WT/WT/**MT**/WT | 5 |
| WT/WT/**MT**/WT/WT/WT | 5 |
| **MT**/**MT**/WT/**MT**/WT/**MT** | 3 |
| **MT**/**MT**/WT/**MT**/WT/WT | 3 |
| WT/WT/WT/**MT**/WT/**MT** | 2 |
| WT/WT/WT/WT/**MT**/**MT** | 2 |

Data are the number of subjects with the haplotype

**SUPPLEMENTAL FIGURES**

**Supplemental Figure 1.** Representation of the best subset of variables provided from the branch-and-bound algorithm of the leaps analysis and distribution of the predictor coefficients for estimated glomerular filtration rate (eGFR) 3 (A), 6 (B) and 12 (C) months after transplantation and for eGFR measured at the time of the exploration visit (D) after 1000 bootstrap replications. Detailed methods used are described in the statistical analysis section.

**Supplemental Figure 2.** Impact of CYP2C9*3 polymorphism on estimated glomerular filtration rate (eGFR) measured at the time of the exploration visit.


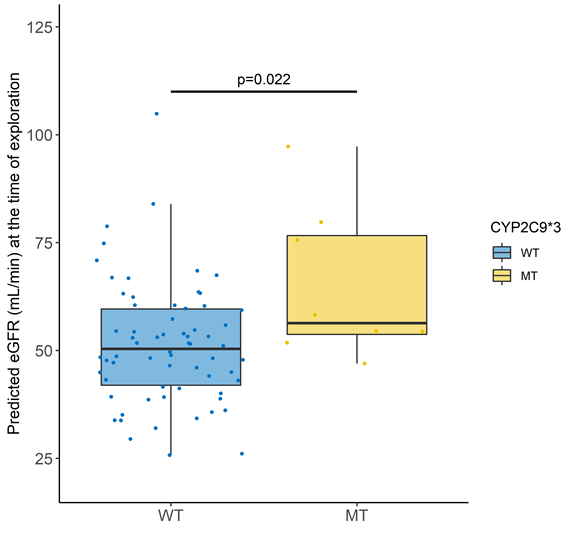


**Supplemental Figure 3.** Representation of the best subset of variables provided from the branch-and-bound algorithm of the leaps analysis and distribution of the predictor coefficients for radial artery flow-mediated dilatation (A), and GTN-induced dilatation (B) after 1000 bootstrap replications. Detailed methods used are described in the statistical analysis section.

**Supplemental Figure 4.** Impact of 3’UTR, rs6558004 and K55R polymorphisms on the baseline levels of each epoxyeicosatrienoic acid (EET) regioisomer.


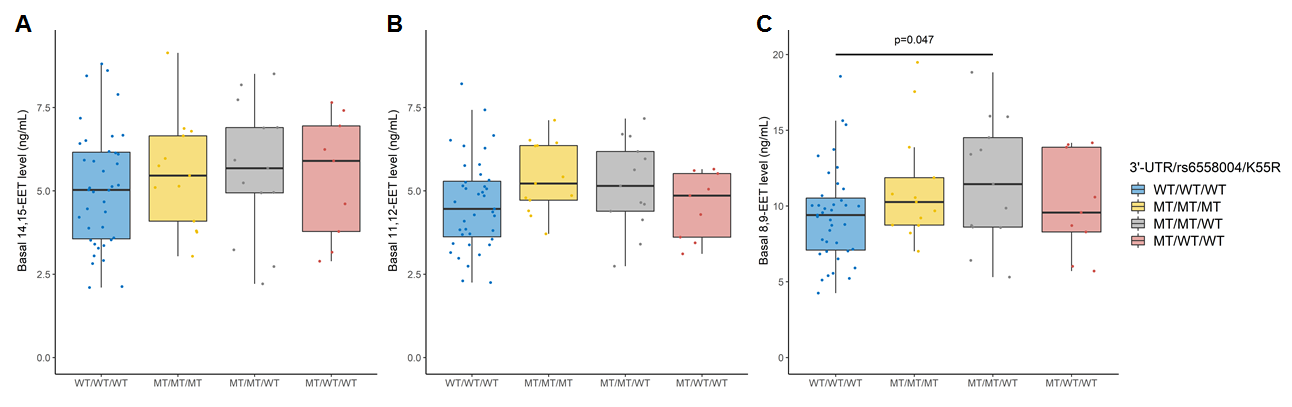


**Supplemental Figure 5.** Impact of R287Q polymorphism on soluble epoxide hydrolase (sEH) activity in isolated peripheral blood mononuclear cells (A), baseline plasma levels of epoxyeicosatrienoic acids (EETs; B) and dihydroxyeicosatrienoic acids (DHETs; C) determined at the time of the exploration visit in kidney transplant recipients.

**Supplemental Figure 6.** Impact of CYP2C9*3, CYP2C8*3/2C9*2 and CYP2C19*17 polymorphisms on baseline plasma levels of epoxyeicosatrienoic acids (EETs; A to C) and dihydroxyeicosatrienoic acids (DHETs; D to F).


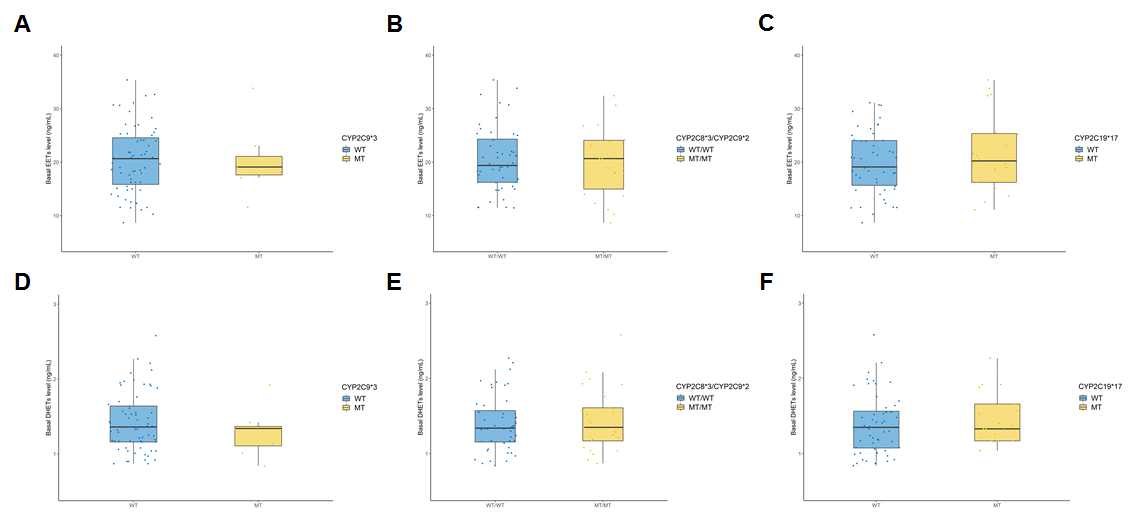

Supplement: Supplementary file 1 — Supplementary Information 1. [file 41598_2021_83274_MOESM1_ESM.docx]
